# Interaction of Structural Glycoprotein E2 of Classical Swine Fever Virus with Protein Phosphatase 1 Catalytic Subunit Beta (PPP1CB)

**DOI:** 10.3390/v11040307

**Published:** 2019-03-29

**Authors:** Elizabeth A. Vuono, Elizabeth Ramirez-Medina, Lauren G. Holinka, Ryan Baker-Branstetter, Manuel V. Borca, Douglas P. Gladue

**Affiliations:** 1 Plum Island Animal Disease Center, ARS, USDA, Greenport, NY 11944, USA; Elizabeth.Vuono2@ars.usda.gov (E.A.V.); Elizabeth.Ramirez@ars.usda.gov (E.R.-M.); Lauren.Holinka-patterson@jax.org (L.G.H.); Ryan.Baker-branstetter@jhuapl.edu (R.B.-B.); 2 Oak Ridge Institute for Science and Education (ORISE), Oak Ridge, TN 37830, USA; 3 Department of Pathobiology and Veterinary Science, University of Connecticut, Storrs, CT 06269, USA

**Keywords:** E2, classical swine fever virus, CSFV, Viral-Host protein interactions

## Abstract

Classical swine fever virus (CSFV) E2 protein, the major virus structural glycoprotein, is an essential component of the viral envelope. E2 is involved in virus absorption, induction of a protective immune response and is critical for virulence in swine. Using the yeast two-hybrid system, we identified protein phosphatase 1 catalytic subunit beta (PPP1CB), which is part of the Protein Phosphatase 1 (PP1) complex, as a specific binding host partner for E2. We further confirmed the occurrence of this interaction in CSFV-infected swine cells by using two independent methodologies: Co-immunoprecipitation and Proximity Ligation Assay. In addition, we demonstrated that pharmacological activation of the PP1 pathway has a negative effect on CSFV replication while inhibition of the PP1 pathway or knockdown of PPP1CB by siRNA had no observed effect. Overall, our data suggests that the CSFV E2 and PPP1CB protein interact in infected cells, and that activation of the PP1 pathway decreases virus replication.

## 1. Introduction

Classical Swine Fever (CSF) is a highly contagious disease of swine that is endemic in most of Asia, Central and South America, and parts of Europe and Africa. Recently the disease has caused an outbreak in Japan [1] after twenty-five years of being CSF free. The etiological agent of this disease, Classical Swine Fever Virus (CSFV), is a small, enveloped virus possessing a positive-stranded RNA genome. CSFV, along with Bovine Viral Diarrhea Virus (BVDV) and Border Disease Virus (BDV), are classified as members of the *Pestivirus* genus within the family *Flaviviridae* [2]. The CSFV genome is 12.5 kb containing a single open reading frame that encodes a 3898-amino-acid polyprotein that yields 11 to 12 final cleavage proteins (NH_2_-Npro-C-E^rns^-E1-E2-p7-NS2-NS3-NS4A-NS4B-NS5A-NS5B-COOH) that are cleaved by processing of the polyprotein by viral and cellular proteases [3]. The CSFV virion contains several structural components, the Core protein and three glycoproteins E^rns^, E1 and E2, which are structurally associated with the virus envelope. The functional significance of the glycoproteins, particularly in the processes of virus replication and virulence have been studied in some detail [4,5,6,7,8,9,10,11].

The host protein partners involved in CSFV infection have recently started to be characterized, with several proteins being shown to specifically interact with structural CSFV proteins. For example, CSFV Core protein has been shown to interact with SUMO1 (small ubiquitin-related modifier 1), IQGAP1 (IQ motif containing GTPase activating protein 1), UBC9 (Ubiquitin Conjugating enzyme 9) and HB (Hemoglobin subunit beta) [12,13,14,15], and E^rns^ has been shown to interact with the Laminin receptor [16]. Similarly, E2 has been identified as an interacting partner with several different host proteins including cellular β-actin [17], Anx2 (Annexin 2) [18], Trx2 (Thioredoxin [19], and MEK2 (mitogen-activated protein kinase 2) [20]. In most of these cases, these host-virus protein interactions play a role in regulating the virus replication cycle.

Previously, we also have identified several host proteins that interact with CSFV E2 using a yeast two-hybrid approach [21]. Here we expanded our previous yeast two-hybrid studies to the identification of additional host factors interacting with the E2 protein, and identified host protein PPP1CB (protein phosphatase 1 catalytic subunit β) as a specific protein binding partner for E2. PPP1CB is part of the highly conserved eukaryotic protein complex protein phosphatase 1 (PP1), a protein serine/threonine phosphatase. Each functional PP1 enzyme has both catalytic subunits and regulatory subunits; the catalytic subunit is comprised of three different subunits PP1α, PP1β and PP1γ. The catalytic subunit associates with a range of host regulatory polypeptides to form a multimeric holoenzyme with distinct substrate specificity of the phosphatase. These regulatory subunits can bind by short degenerate sequence motifs, typically 4–6 residues in lengths, with most regulatory subunits having multiple points of interaction with PP1 catalytic subunits. One of the regulatory binding sites of PP1 has been mapped and is typically referred to as the RVxF binding channel available at: https://www.physiology.org/doi/full/10.1152/physrev.00013.2003. This binding channel often serves as an anchor for the initial binding to PP1, often-promoting binding to other binding sites [22,23].

Viruses in general have evolved to evade the immune system by various methods and inhibiting the anti-viral activity of PP1 is among them. For example, Hepatitis B virus (HBV) protein HBx has been determined to directly bind and neutralize PP1 to enhance virus transcription [24]. Measles Virus (MV) has two mechanisms that inhibits cellular PP1 activity by suppressing MDA5 (Melanoma Differentiation-Associated protein 5) and RIG-I (retinoic acid-inducible gene I) upstream activators of PP1 [25]. MV protein V and Nipah virus protein V have been determined to inhibit dephosphorylation of MDA5 by directly binding PP1α/γ subunits [26]. Respiratory syncytial virus (RSV) protein P directly binds PP1 to dephosphorylate viral protein M2-1, a viral transcription factor, necessary to transcribe viral mRNA [27]. A similar mechanism was observed in the Ebola virus where PP1 dephosphorylates VP30 [28] and also in HIV where the Tat protein binds PP1 to regulate viral transcription [29].

In this report, we determine that host protein PPP1CB specifically interacts with the CSFV E2 protein. We identified the PPP1CB-E2 interaction by yeast two-hybrid and further confirmed that this protein–protein interaction occurs during CSFV infection in swine cells by co-immunoprecipitation assays (CIPA) and by a proximity ligation assay (PLA). We demonstrated that pharmacological activation of the PP1 pathway had a negative effect on CSFV replication while inhibition of the PP1 pathway or knockdown of PPP1CB by siRNA had no observed effect. We also identified in the amino acid sequence of CSFV E2 protein a PP1 recognition motif and several predicted phosphorylation sites, suggesting that E2 is phosphorylated by PP1. Utilizing a PLA we were able to determine close association between phosphorylation and CSFV E2 suggesting that CSFV E2 is phosphorylated, at least transiently during viral infection.

## 2. Materials and Methods

### 2.1. Cells and Virus

CSFV strain Brescia (BICv) is a derivative obtained by transfection of the Brescia infectious clone (pBIC) in SK6 cells as described elsewhere [5]. Virus stocks were prepared in swine kidney cells (SK6). SK6 cells, free of BVDV, were grown in Dulbecco’s Minimal Essential Media (DMEM) (Gibco, Grand Island, NY, USA) using 10% fetal calf serum (FCS) (Atlas Biologicals, Fort Collins, CO, USA). Virus growth kinetics were evaluated on primary swine macrophage cell cultures, which were prepared as previously described [30]. Titration of CSFV was performed using the Reed and Muench methodology [31] expressed as TCID_50_/mL with sensitivity of ≥1.8 TCID_50_/mL. In brief, SK6 cells were plated in 96-well plates (Costar, Cambridge, MA, USA) and at 4 days post-infection (dpi) the presence of infectious virus was determined by immunostaining using anti-E2 CSFV monoclonal antibody WH303 [32].

### 2.2. Yeast Two-Hybrid Screening

We used a yeast two-hybrid screening approach using a custom swine macrophage library previously described in more detail [21]. In brief, a yeast two-hybrid screening approach to identify novel host cellular proteins that interact with CSFV E2 protein. An N-terminal fusion of the Gal4 protein DNA binding domain (BD) with CSFV E2 protein was used as “bait.” For “prey,” our custom cDNA library that was derived from swine macrophage RNA and expressed as N-terminal fusions to the Gal4 activation domain (AD). Screening was conducted with positive protein interactions showing growth on yeast growth plates that lack histidine. Plasmid DNA was isolated from positive colonies, sequenced and retested for the ability of the AD plasmid to interact with E2 but not with a negative control.

### 2.3. Immunoblotting and Antibodies

For immunoblotting analysis infected cell monolayers or media only control (mock infected) were washed in ice-cold PBS and lysed in RIPA Buffer (Teknova, Hollister, CA, USA) in the presence of a protease inhibitor cocktail (Roche, Basel, Switzerland). Proteins were resolved on NuPAGE 4–12% *w*/*v* Bis-Tris gels (Invitrogen, Carlsbad, CA, USA) and transferred to polyvinylidene difluoride (PVDF) membranes following manufacturer instructions. Immunodetection was performed using the following antibodies: Rabbit polyclonal Anti-PPP1CB using a dilution of 1:1000 (Cat# ab53315, Abcam, Cambridge, UK), mouse monoclonal antibody anti-α-Tubulin using a dilution of 1:3000 DM1A (Thermo Fisher Scientific, Waltham, MA, USA), and anti-CSFV E2 protein monoclonal antibody WH303 using a dilution of 1:1000 [32] and Pierce Goat Anti-Rabbit IgG peroxidase conjugated or Pierce Goat Anti-Mouse IgG peroxidase conjugated secondary antibody reagent (Thermo Fisher Scientific, Waltham, MA, USA). Western blots were imaged using an Azure C300 and analyzed with cSeries capture software (Azure Biosystems, Dublin, CA, USA).

### 2.4. Immunoprecipitation

Immunoprecipitation procedures were performed in triplicate using the Pierce Co-immunoprecipitation kit following manufacturer’s instructions (Thermo Fisher Scientific). In brief, cell suspensions were washed in ice-cold PBS and lysed with the Pierce Lysis buffer plus protease inhibitors cocktail (Roche, Basel, Switzerland). Anti-E2 WH303 [32] was conjugated to Pierce beads and incubated with precleared lysate overnight. The beads were then washed with Pierce Lysis buffer plus 0.1% Triton-X-100 (Sigma-Aldrich, St. Louis MO, USA) and protease inhibitors cocktail (Roche) and eluted in Pierce elution buffer.

### 2.5. Proximity Ligation Assay

The PLA was performed in triplicate using guidelines from the Duolink-PLA kit (Sigma-Aldrich). SK6 cells were plated onto 12 mm round coverslips (Thomas Scientific, Swedesboro, NJ, USA) in a 24-well plate (Corning, Corning, NY, USA) at a density of 25,000 cells/well. The following day cells were infected at multiplicity of infection (MOI) = 10 and 24 h later, cells were fixed with 4% formaldehyde *w*/*v* in PBS at room temperature for 20 min, followed by permeabilization buffer (0.3% Triton-X-100 in PBS) for 10 min. Fixed cells were then blocked with Duolink blocking buffer for 30 min at 37 °C followed by incubation with primary antibodies Anti-E2 WH303 using a dilution of 1:1000 [32] and Anti-PP1β using a dilution of 1:1000 (Abcam cat# ab53315) or Anti-pan pS/T using a dilution of 1:1000 (Abcam cat# ab117253) at 4 °C for 1 h. The cells were then washed 2 times with 1× Duolink (Sigma-Aldrich) wash buffer A and incubated with the PLUS and MINUS PLA probes for 1 h at 37 °C followed by 2 washes with 1× Duolink wash buffer A and 30 min incubation at 37 °C with Duolink Ligase in 1× ligation buffer. The fixed cells were then washed twice with 1× Duolink wash buffer and followed by incubation with Duolink polymerase in 1× Amplification buffer at 37 °C for 100 min. The fixed cells were then washed twice with Duolink wash buffer B and mounted with Duolink PLA Mounting Medium with DAPI.

### 2.6. PP1 Activating and Inhibitory Experiments

Cells were plated in a six-well dish at a density of 500,000 cells/well. The following day cells were treated with PP1 inhibitor (OA) at 5 nM or PP1 activator ceramide (C6) at 20 µM for 1 h prior to infection with CSFV at a MOI = 1 for 2 h. Cells were then washed twice with PBS and replaced with media containing either 5 nM OA or 20 µM C6. Time points were collected at 0 and 24 h post infection (hpi) and virus yield quantified by titration. P values were calculated using a standard *t*-test.

### 2.7. siRNA Experiments

Cells were plated in a six-well dish at a density of 200,000 cells/well and incubated overnight at 37 °C in the presence of 5% CO_2_. Cells were transfected using Lipofectamine 2000 (Invitrogen) with PPP1CB-specific (siPPP1CB), or as controls, non-targeting siRNA (siCtrl), lipofectamine-treated cells or untreated cells for 24 h. Then cells were infected with CSFV at a MOI = 0.1 for 1 h, inoculum was removed and replaced with growth media. Time points were then collected at 0 and 24 hpi. The following sense and anti-sense siRNA sequences were used; siPPP1CB (Dharmacon, Lafayette, CO, USA) 5′-GAAGAUUUGUGGAGAUAUUUU-3′ and 5′-AAUAUCUCCACAAAUCUUCUU-3′ and siGENOME non-targeting siRNA pool #2 (Dharmacon D-001206-14-05). The *p* values were calculated using a standard *t*-test.

## 3. Results

### 3.1. CSFV Structural E2 Protein Binds Swine PPP1CB

We use a previously described [21] yeast two-hybrid system along with a custom swine macrophage library to identify novel host cellular proteins that interact with CSFV E2 protein. An N-terminal fusion of the Gal4 protein DNA binding domain (BD) with CSFV E2 protein was used as “bait.” For “prey,” we used a custom cDNA library that was derived from swine macrophage RNA and expressed as N-terminal fusions to the Gal4 activation domain (AD). The screening identified swine PPP1CB (NCBI Gene ID# 397378) as a specific binding partner for CSFV E2. To address the issue of false-positive interaction with CSFV E2 protein, human lamin C protein (BD-LAM) was used as a negative control (Table 1) demonstrating that host PPP1CB and virus E2 specifically interacts as detected by yeast two-hybrid methodology.

Previously, we constructed a set of 76 CSFV E2 mutant proteins, utilizing an alanine scanning mutagenesis approach, to design a library of E2 mutants with substitutions of sequential stretches of the native amino acids for alanine [21]. Similar to our previous results obtained with other cellular proteins that were identified as binding partners for E2 [21], we were unable to determine a binding site for PPP1CB using this set of mutants suggesting that a single linear stretch of amino acids substituted for alanine was not sufficient in disrupting the binding site for PPP1CB. This could indicate that the E2 binding site for PPP1CB is non-linear or the presence of multiple binding sites exist in E2.

### 3.2. CSFV E2 Protein Interacts with Host PPP1CB during the Virus Infectious Cycle

**Figure 1:**
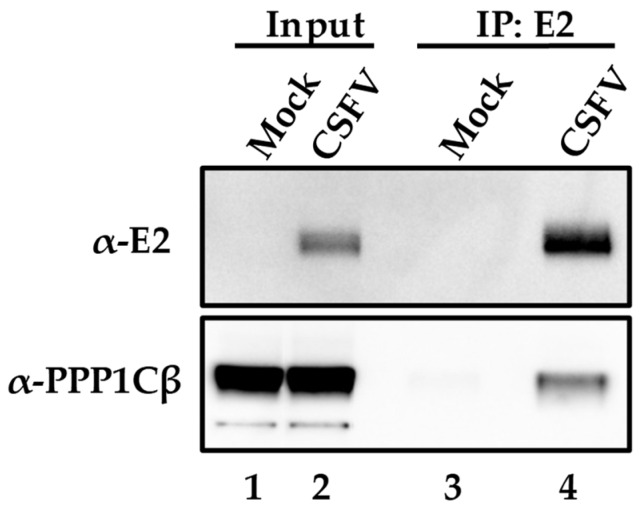
Co-immunoprecipitation of E2 in CSFV-infected or mock-infected cells. The input cell lysate is in lanes 1 and 2 and the lysate that was immunoprecipitated with antibodies for E2 in lanes 3 and 4. Protein extracts were blotted for PPP1BC and E2 as indicated in Materials and Methods with bands observed at the expected molecular weights of 55kDa for E2 and 37 kDa for PPP1CB.

To further confirm the PPP1CB-E2 protein interaction discovered using the two-hybrid system in yeast actually occurs during CSFV infection of host cells, co-immunoprecipitation experiments were performed using monoclonal antibodies (mAbs) specifically recognizing both interacting proteins. SK6 cells were infected (MOI = 1) with CSFV isolate Brescia, a highly pathogenic strain of CSFV [5]. Samples were harvested at 24 h post-infection (hpi) and processed as described in Materials and Methods. SK6 cell lysates were collected from infected or mock-infected cells and immunoprecipitated with an anti-E2 mAb, WH303 [32], followed by a Western blot with a PPP1CB-specific mAb, (Abcam). A single band at the expected molecular mass of PPP1CB (37 kDa) was clearly observed indicating that in CSFV-infected cells E2 can be co-immunoprecipitated along with PPP1CB. This result confirms the previous yeast two-hybrid finding, suggesting E2-PPP1CB interaction occurs during viral infection of cell cultures (Figure 1).

**Figure 2:**
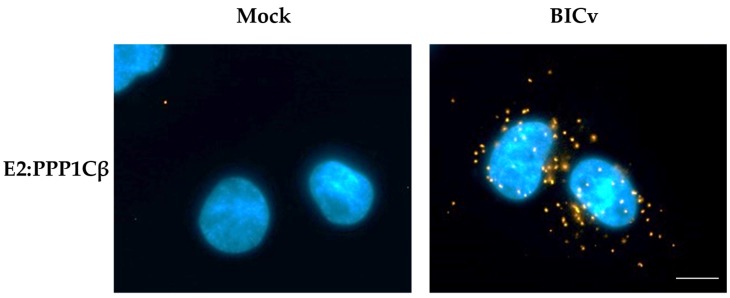
PLA for E2 and PPP1CB in CSFV infected SK6 cells. Interaction between E2 and PPP1CB was determined by PLA in SK6 cells that were either mock-infected or infected for 24 h with CSFV BICv (MOI = 10). Scale bar is 50 µM.

During infection with CSFV, E2 is highly abundant throughout the cell cytoplasm, and consequently, it is hard to easily visualize the potential subcellular co-localization of E2 along with cellular proteins. Proximity ligation assays [33] have the advantage of allowing for the direct identification of transient protein–protein interactions, without the additional background observed in classical co-localization studies. Detection of proteins E2 and PPP1CB were performed using mAb WH303 [32] for detection of E2 and a commercial mAb (Abcam # ab53315) for detection of PPP1CB. Results of the proximity ligation assay confirm that E2 and PPP1CB interact in SK6 cell cultures infected with CSFV, and that this interaction, analyzed at 24 hpi (under similar conditions as those used to perform the co-immunoprecipitation studies), occurs in distinct punctate locations throughout the cell cytoplasm (Figure 2). Therefore, both methodologies (co-immunoprecipitation and proximity ligation assay) confirmed the existence of E2 and PP1 interaction during the CSFV infection of SK6 cell cultures.

### 3.3. CSFV Is Transiently Phosphorylated during Virus Infection

**Figure 3:**
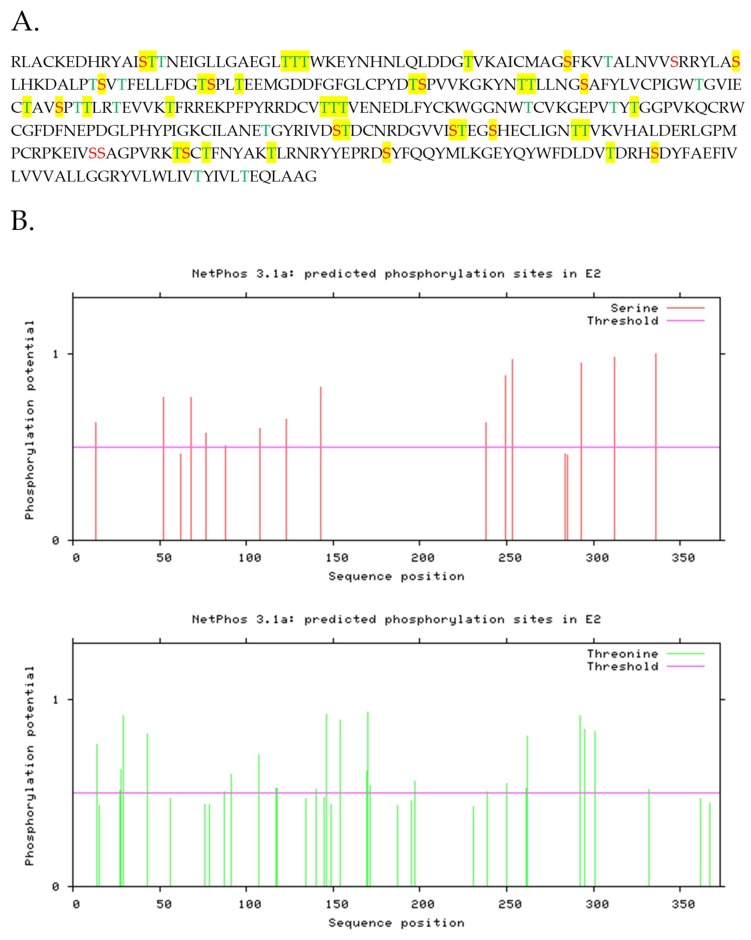
Prediction of phosphorylation sites in CSFV E2 (**A**) Amino acid sequence of CSFV E2 with serine displayed in red and threonine displayed in green. Highlighted residues indicate residues that are predicted to be phosphorylated. (**B**) Prediction in NetPhos displaying the phosphorylation potential.

Interaction between CSFV E2 and PP1 may indicate that E2 could be phosphorylated during the virus replication cycle, a fact that has not been previously described. In silico analysis of CSFV E2 using NetPhos 3.1 server [34] was used to predict the presence of potential serine or threonine phosphorylation sites. The server predicted the presence of 13 serine sites (at E2 amino acid positions 13, 52, 68, 77, 88, 108, 123, 143, 238, 249, 293, 312, and 336) and 24 Threonine sites (at E2 amino acid positions 14, 27, 28, 29, 43, 87, 91, 107, 118, 140, 146, 154, 169, 170, 171, 197, 239, 250, 261, 262, 292, 295, 301, and 332) that could be functioning as possible phosphorylation sites (Figure 3A). The predicted sites are highlighted in the amino acid sequence (Figure 3B) and showed that the predicted phosphorylation sites are throughout the coding sequence of E2, increasing the likelihood that E2 is phosphorylated during viral infection.

**Figure 4:**
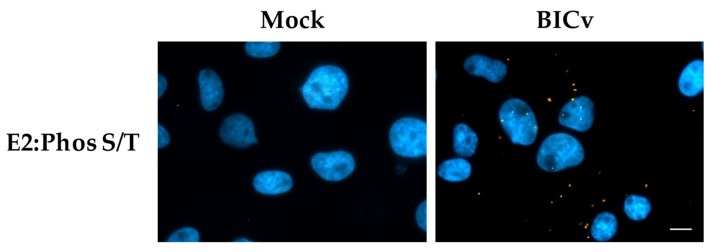
Detection of E2 phosphorylation by PLA. Phosphorylation of E2 was determined with PLA using a pan phosphor-serine/threonine and E2 antibody in SK6 cells either mock infected or with CSFV BICv (MOI = 10). Scale bar is 50 µM.

To determine if E2 is actually phosphorylated during infection in swine cells we utilized the PLA using monoclonal antibody WH303 to recognize E2 along with an anti-phosphorylated serine/threonine antibody that specifically recognizes the presence of phosphorylated serine or threonine residues (Abcam ab117253). This methodology has been widely used to determine when a protein is phosphorylated [35]. Our results were positive in PLA and demonstrated that E2 is phosphorylated in infected SK6 cells when tested at 24 hpi (Figure 4).

### 3.4. Pharmacological Activation of PP1 Inhibits CSFV Replication

**Figure 5:**
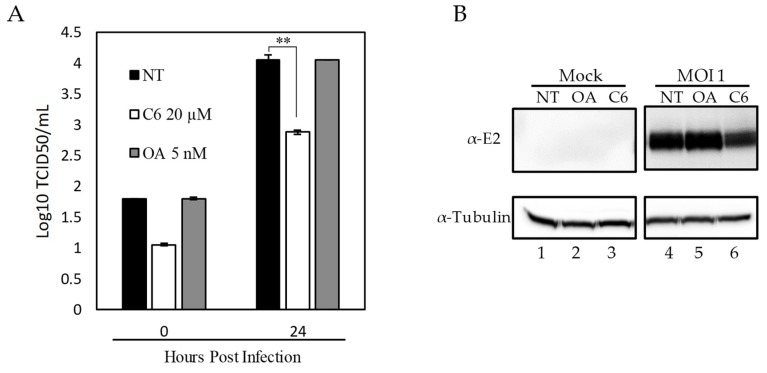
Effect of PPP1CB phosphatase activity on CSFV replication. SK6 cells were pretreated with no drug, PPP1CB phosphatase inhibitor Okadaic Acid (OA), phosphatase activator Ceramide (C6) or no treatment (NT) followed by infection with CSFV (MOI:1) or mock for 2 h. Time points were collected 0 and 24 h post infection (hpi). (**A**) Viral yields from CSFV infection in SK6 cells treated as indicated. Titers were determined in SK6 cells and expressed as the log 10 TCID 50 /mL, +/−standard error from triplicate samples (OA: Okadaic Acid, C6: Ceramide) ** *p* Value: <0.01). (**B**) Whole-cell lysates were collected 24 hpi. Immunoblotting with an E2-specific antibody (band is 55 kDa as expected) showed a decrease in E2 protein concentration with C6 treatment compared to OA and NT (lane 6 vs. 4 and 5). An α-Tubulin-specific antibody was used as a loading control (band is at 50 kDa as expected).

To evaluate the role of PP1 activity in CSFV replication we utilized the pharmacological activator of PP1, Ceramide (C6) [36], and the inhibitor of PP1, Okadaic Acid (OA) [37]. Chemical modulators were used at concentrations previously described for OA [37] and for C6 [36]. At these concentrations, there was no observed drug-induced toxicity in SK6 cells at 48 h post-treatment. SK6 cells (5 × 10^6^ cells/well) were plated in 6-well plates and the following day cells were treated with either 5 nM (OA) or 20 µM of C6 one hour prior to CSFV infection. Cells were then infected at a MOI = 1 for 2 h, and the virus inoculum was removed. Cells were incubated at 37 °C in the presence of the corresponding drug with samples collected at 24 hpi, and virus yield quantified by titration in SK6 cells. Results demonstrated that no significant differences in virus yields were observed when the PP1 pathway was inhibited by using OA. Conversely, virus replication significantly decreased, by approximately 10-fold, when the PP1 pathway was activated by treatment with C6 (Figure 5). To determine if this reduction in viral yields was due to a decrease in viral protein or a decrease in infectious virus, samples were taken at 24 hpi and protein extracts were determined by Western blot. Again, no observed change in viral protein expression was observed using OA, while a decrease in viral protein was observed in cells that were treated with C6. These results (Figure 5) suggest that activation of the PP1 pathway negatively affect both CSFV protein production and viral replication.

### 3.5. siRNA Knockdown of Cellular PPP1CB

**Figure 6:**
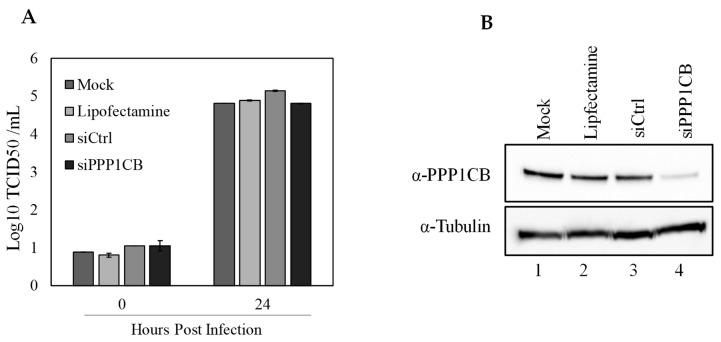
Effect of PPP1CB-specific siRNA treatment on CSFV replication. SK6 cells were transfected with either siRNA targeting PPP1CB, a control siRNA (siCtrl), lipofectamine alone or left untreated (mock). Cells were infected with CSFV MOI 0.1 (**A**) Viral yields from CSFV infected SK6 cells treated as indicated. Supernatants were collected at 0 and 24 hpi and titrated in SK6 cells and expressed as log_10_ TCID50/mL +/−standard error from triplicate samples. (**B**) Whole-cell lysates were collected 48 h after siRNA treatment and analyzed by immunoblotting with a PPP1CB-specific antibody (band is 37 kDa) or with an α-Tubulin-specific antibody (band is at 50 kDa).

To further understand the role for PPP1CB during CSFV infection, we utilized a specific siRNA that will prevent PPP1CB from being translated and reduce cellular levels of PPP1CB to evaluate if decreasing cellular PPP1CB will have an effect on CSFV replication. SK6 cells were pretreated with siRNA for 24 h and infected with CSFV at an MOI of 0.1. Twenty-four hpi supernatant samples were taken for titration, and cell lysate was assessed by Western blotting to determine the levels of PPP1CB in the cell lysate. It was clear that the specific siRNA had a decrease in PPP1CB when compared to siRNA controls, or untreated cells. However, there was no evident decrease in viral titers between the treatments, suggesting that cells with a reduced level of PPP1CB did not have an effect on virus replication (Figure 6).

## 4. Discussion

Regulation of cellular phosphorylation by PP1 occurs in a variety of cellular pathways and processes, and is a complex cellular regulatory mechanism. The catalytic subunits of PP1 bind a PP1 regulatory subunit in which there are over 200 different proteins; this large number of PP1 regulatory subunits demonstrates the wide range of serine/threonine phosphatase substrates [38]. Some of the cellular processes regulated by PP1 are involved in antiviral activity, creating a pathway that is often targeted for disruption by invading viruses. For example, cystolic sensors for viral RNA such as MDA5 or RIG-I are activated by PP1 dephosphorylation, and both belong to the family of RLRs. Their activation results in a signaling cascade that results in activation of host antiviral pathways. In order to evade this antiviral effect several viruses have evolved to dysregulate the activity of PP1. Several viruses have been previously determined to directly bind PP1 catalytic subunits, similar to what was observed in this report. For example, HIV Tat protein directly binds PP1α, regulating its activity in mouse neurons, suggesting that the Tat-PP1α protein interaction could be the cause of impaired cognitive function in people living with HIV [39]. Several studies have also shown that inhibitors of PP1, such as small molecules that bind RVxF motifs, have inhibited HIV viral transcription [39]. On the other hand, some viruses require an active PP1 pathway. In the Rift Valley Fever virus (RVFV), PP1 is necessary for viral replication, as inhibition of PP1 activity causes a decrease in early viral replication in cell cultures [40]. Similar results with the Ebola virus (EBOV) have been shown that EBOV protein VP30 phosphorylation is controlled by PP1 and phosphorylation of VP30 causes inhibition of viral transcription [28].

Here we describe a specific interaction between the cellular PPP1CB protein and the CSFV E2 glycoprotein. We were able to determine that this interaction is specific using a yeast two-hybrid system, and further confirmed this interaction in virally infected cells by using both co-immunoprecipitation and PLA techniques. Further, we determined that E2 contains several potential phosphorylation sites and, by using PLA, that E2 is at least transiently phosphorylated. Pharmacological manipulation of the PP1 pathway by using a well-characterized activator C6 and an inhibitor OA determined that PP1 activation decreased CSFV replication in treated SK6 cell cultures. This result suggests that the PP1 pathway is not required for CSFV transcription, as observed in some other viruses, but rather virus replication is inhibited when the PP1 pathway is activated. It is possible that the interaction of E2 with PPP1CB inhibits or selectively inhibits dephosphorylation caused by PP1, possibly to circumvent the antiviral response caused by PP1. Activating the PP1 pathway could counter this effect and allow the cell to overcome the PP1 inhibition induced by E2 and could be the reason why in this study activation of PP1 caused a decrease in CSFV replication in swine cell cultures. It is also possible that the transient phosphorylation of CSFV E2 is necessary for an undetermined function of E2, or simply a mechanism to bind and inhibit PP1 to prevent PP1 from dephosphorylating other cellular downstream effectors that have a negative effect on CSFV replication. Further we show using siRNA that decreasing cellular levels of PPP1CB did not alter CSFV replication, indicating that either the activity of PPP1CB is not required or the residual levels of PP1CB for CSFV replication in cell cultures.

PP1 has been shown to be involved in host immune sensing of viruses, where dephosphorylation of several sensing proteins leads to activation of antiviral pathways. For example in the Measles Virus, protein V antagonizes PP1, to prevent the removal of the phosphorylation and activation of MDA5, a pattern recognition receptor that specifically recognizes dsRNA, as part of the antiviral response. It has been previously shown that CSFV can be sensed by MDA-5 [41] and that activation of MDA5 by OASL (2′-5′-oligoadenylate synthetase-like protein) had antiviral effects on CSFV [42]. It is possible that binding of PP1 by E2 could be a mechanism in which CSFV evades the host antiviral response by preventing dephosphorylation and thus activation of antiviral pathways. Although we were unable to determine the exact role for E2 to bind PP1β, we did show that E2 is phosphorylated, and it is possible that binding of PP1β would inhibit the PP1 complex from dephosphorylating and activating host antiviral proteins. However, we did determine that the PP1 pathway does have an effect on CSFV replication, since virus replication decreased when the PP1 pathway was activated. Further studies will be needed to determine the specific effects that CSFV may achieve by manipulating the host PP1 pathway.

In summary, this report allowed us to gain insight into a novel host-viral protein interaction between CSFV E2 and host PPP1CB. This study, along with previous reports by us and others, demonstrate that the E2 protein plays a central role at several stages of the virus life cycle. The many functions of E2 are likely to be mediated by post-translational modifications, or by direct interaction with host proteins; these functions are likely to have significant impact during viral infection in vitro and in vivo. Identification and understanding of the many host-virus protein interactions is necessary to understand the molecular mechanisms of viral replication and virulence. It is necessary to understand these pathways and protein players involved in order to guide the potential development of better countermeasures to control CSFV infection in swine.

## Figures and Tables

**Table 1:** Protein–protein interaction between the indicated AD and BD plasmids in yeast.

	E2-BD	LAM-BD	BD
PPP1CB-AD	+++	-	-
AD	-	-	-

## References

[1] Nishi T., Kameyama K.I., Kato T., Fukai K. (2019). Genome Sequence of a Classical Swine Fever Virus of Subgenotype 2.1, Isolated from a Pig in Japan in 2018. Microbiol. Resour. Announc..

[2] Fauquet C.M., Fargette D. (2005). International Committee on Taxonomy of Viruses and the 3,142 unassigned species. Virol. J..

[3] Rice C.M., Fields B.N., Knipe D.M., Howley P.M. (1996). Flaviviridae: The Viruses and Their Replication. Fundamental Virology.

[4] Meyers G., Saalmuller A., Buttner M. (1999). Mutations abrogating the RNase activity in glycoprotein E(rns) of the pestivirus classical swine fever virus lead to virus attenuation. J. Virol..

[5] Risatti G.R., Borca M.V., Kutish G.F., Lu Z., Holinka L.G., French R.A., Tulman E.R., Rock D.L. (2005). The E2 glycoprotein of classical swine fever virus is a virulence determinant in swine. J. Virol..

[6] Risatti G.R., Holinka L.G., Carrillo C., Kutish G.F., Lu Z., Tulman E.R., Sainz I.F., Borca M.V. (2006). Identification of a novel virulence determinant within the E2 structural glycoprotein of classical swine fever virus. Virology.

[7] Risatti G.R., Holinka L.G., Fernandez Sainz I., Carrillo C., Kutish G.F., Lu Z., Zhu J., Rock D.L., Borca M.V. (2007). Mutations in the carboxyl terminal region of E2 glycoprotein of classical swine fever virus are responsible for viral attenuation in swine. Virology.

[8] Risatti G.R., Holinka L.G., Fernandez Sainz I., Carrillo C., Lu Z., Borca M.V. (2007). N-linked glycosylation status of classical swine fever virus strain Brescia E2 glycoprotein influences virulence in swine. J. Virol..

[9] Risatti G.R., Holinka L.G., Lu Z., Kutish G.F., Tulman E.R., French R.A., Sur J.H., Rock D.L., Borca M.V. (2005). Mutation of E1 glycoprotein of classical swine fever virus affects viral virulence in swine. Virology.

[10] van Rijn P.A., Miedema G.K., Wensvoort G., van Gennip H.G., Moormann R.J. (1994). Antigenic structure of envelope glycoprotein E1 of hog cholera virus. J. Virol..

[11] Borca M.V., Holinka L.G., Ramirez-Medina E., Risatti G.R., Vuono E.A., Berggren K.A., Gladue D.P. (2019). Identification of structural glycoprotein E2 domain critical to mediate replication of Classical Swine Fever Virus in SK6 cells. Virology.

[12] Li D., Dong H., Li S., Munir M., Chen J., Luo Y., Sun Y., Liu L., Qiu H.J. (2013). Hemoglobin subunit beta interacts with the capsid protein and antagonizes the growth of classical swine fever virus. J. Virol..

[13] Gladue D.P., Holinka L.G., Fernandez-Sainz I.J., Prarat M.V., O’Donell V., Vepkhvadze N., Lu Z., Rogers K., Risatti G.R., Borca M.V. (2010). Effects of the interactions of classical swine fever virus Core protein with proteins of the SUMOylation pathway on virulence in swine. Virology.

[14] Gladue D.P., Holinka L.G., Fernandez-Sainz I.J., Prarat M.V., O’Donnell V., Vepkhvadze N.G., Lu Z., Risatti G.R., Borca M.V. (2011). Interaction between Core protein of classical swine fever virus with cellular IQGAP1 protein appears essential for virulence in swine. Virology.

[15] Gladue D.P., O’Donnell V., Fernandez-Sainz I.J., Fletcher P., Baker-Branstetter R., Holinka L.G., Sanford B., Carlson J., Lu Z., Borca M.V. (2014). Interaction of structural core protein of classical swine fever virus with endoplasmic reticulum-associated degradation pathway protein OS9. Virology.

[16] Chen J., He W.R., Shen L., Dong H., Yu J., Wang X., Yu S., Li Y., Li S., Luo Y. (2015). The laminin receptor is a cellular attachment receptor for classical Swine Fever virus. J. Virol..

[17] He F., Ling L., Liao Y., Li S., Han W., Zhao B., Sun Y., Qiu H.J. (2014). Beta-actin interacts with the E2 protein and is involved in the early replication of classical swine fever virus. Virus Res..

[18] Yang Z., Shi Z., Guo H., Qu H., Zhang Y., Tu C. (2015). Annexin 2 is a host protein binding to classical swine fever virus E2 glycoprotein and promoting viral growth in PK-15 cells. Virus Res..

[19] Li S., Wang J., He W.R., Feng S., Li Y., Wang X., Liao Y., Qin H.Y., Li L.F., Dong H. (2015). Thioredoxin 2 Is a Novel E2-Interacting Protein That Inhibits the Replication of Classical Swine Fever Virus. J. Virol..

[20] Wang J., Chen S., Liao Y., Zhang E., Feng S., Yu S., Li L.F., He W.R., Li Y., Luo Y. (2016). Mitogen-activated Protein Kinase Kinase 2 (MEK2), a Novel E2-interacting Protein, Promotes the Growth of Classical Swine Fever Virus via Attenuation of the JAK-STAT Signaling Pathway. J. Virol..

[21] Gladue D.P., Baker-Bransetter R., Holinka L.G., Fernandez-Sainz I.J., O’Donnell V., Fletcher P., Lu Z., Borca M.V. (2014). Interaction of CSFV E2 protein with swine host factors as detected by yeast two-hybrid system. PLoS ONE.

[22] Bollen M. (2001). Combinatorial control of protein phosphatase-1. Trends Biochem. Sci..

[23] Wakula P., Beullens M., Ceulemans H., Stalmans W., Bollen M. (2003). Degeneracy and function of the ubiquitous RVXF motif that mediates binding to protein phosphatase-1. J. Biol. Chem..

[24] Cougot D., Allemand E., Riviere L., Benhenda S., Duroure K., Levillayer F., Muchardt C., Buendia M.A., Neuveut C. (2012). Inhibition of PP1 phosphatase activity by HBx: a mechanism for the activation of hepatitis B virus transcription. Sci. Signal.

[25] Mesman A.W., Zijlstra-Willems E.M., Kaptein T.M., de Swart R.L., Davis M.E., Ludlow M., Duprex W.P., Gack M.U., Gringhuis S.I., Geijtenbeek T.B. (2014). Measles virus suppresses RIG-I-like receptor activation in dendritic cells via DC-SIGN-mediated inhibition of PP1 phosphatases. Cell Host Microbe.

[26] Davis M.E., Wang M.K., Rennick L.J., Full F., Gableske S., Mesman A.W., Gringhuis S.I., Geijtenbeek T.B., Duprex W.P., Gack M.U. (2014). Antagonism of the phosphatase PP1 by the measles virus V protein is required for innate immune escape of MDA5. Cell Host Microbe.

[27] Richard C.A., Rincheval V., Lassoued S., Fix J., Cardone C., Esneau C., Nekhai S., Galloux M., Rameix-Welti M.A., Sizun C. (2018). RSV hijacks cellular protein phosphatase 1 to regulate M2-1 phosphorylation and viral transcription. PLoS Pathog..

[28] Ilinykh P.A., Tigabu B., Ivanov A., Ammosova T., Obukhov Y., Garron T., Kumari N., Kovalskyy D., Platonov M.O., Naumchik V.S. (2014). Role of protein phosphatase 1 in dephosphorylation of Ebola virus VP30 protein and its targeting for the inhibition of viral transcription. J. Biol. Chem..

[29] Ammosova T., Jerebtsova M., Beullens M., Lesage B., Jackson A., Kashanchi F., Southerland W., Gordeuk V.R., Bollen M., Nekhai S. (2005). Nuclear targeting of protein phosphatase-1 by HIV-1 Tat protein. J. Biol. Chem..

[30] Zsak L., Lu Z., Kutish G.F., Neilan J.G., Rock D.L. (1996). An African swine fever virus virulence-associated gene NL-S with similarity to the herpes simplex virus ICP34.5 gene. J. Virol..

[31] Reed L.J., Muench H. (1938). A simple method of estimating fifty percent endpoints. Am. J. Hyg..

[32] Edwards S., Moennig V., Wensvoort G. (1991). The development of an international reference panel of monoclonal antibodies for the differentiation of hog cholera virus from other pestiviruses. Vet. Microbiol..

[33] Soderberg O., Gullberg M., Jarvius M., Ridderstrale K., Leuchowius K.J., Jarvius J., Wester K., Hydbring P., Bahram F., Larsson L.G. (2006). Direct observation of individual endogenous protein complexes in situ by proximity ligation. Nat. Methods.

[34] Blom N., Gammeltoft S., Brunak S. (1999). Sequence and structure-based prediction of eukaryotic protein phosphorylation sites. J. Mol. Biol..

[35] Gullberg M., Andersson A.-C. (2009). Highly specific detection of phosphorylated proteins by Duolink. Nat. Methods.

[36] Chalfant C.E., Kishikawa K., Mumby M.C., Kamibayashi C., Bielawska A., Hannun Y.A. (1999). Long chain ceramides activate protein phosphatase-1 and protein phosphatase-2A. Activation is stereospecific and regulated by phosphatidic acid. J. Biol. Chem..

[37] Zhang S., Sun Y., Chen H., Dai Y., Zhan Y., Yu S., Qiu X., Tan L., Song C., Ding C. (2014). Activation of the PKR/eIF2alpha signaling cascade inhibits replication of Newcastle disease virus. Virol. J..

[38] Bollen M., Peti W., Ragusa M.J., Beullens M. (2010). The extended PP1 toolkit: designed to create specificity. Trends Biochem. Sci..

[39] Liu Y., Zhou D., Feng J., Liu Z., Hu Y., Liu C., Kong X. (2018). HIV-1 Protein Tat1-72 Impairs Neuronal Dendrites via Activation of PP1 and Regulation of the CREB/BDNF Pathway. Virol. Sin..

[40] Baer A., Shafagati N., Benedict A., Ammosova T., Ivanov A., Hakami R.M., Terasaki K., Makino S., Nekhai S., Kehn-Hall K. (2016). Protein Phosphatase-1 regulates Rift Valley fever virus replication. Antiviral Res..

[41] Husser L., Alves M.P., Ruggli N., Summerfield A. (2011). Identification of the role of RIG-I, MDA-5 and TLR3 in sensing RNA viruses in porcine epithelial cells using lentivirus-driven RNA interference. Virus Res..

[42] Li L.F., Yu J., Zhang Y., Yang Q., Li Y., Zhang L., Wang J., Li S., Luo Y., Sun Y. (2017). Interferon-Inducible Oligoadenylate Synthetase-Like Protein Acts as an Antiviral Effector against Classical Swine Fever Virus via the MDA5-Mediated Type I Interferon-Signaling Pathway. J. Virol..

